# A deep learning-based automatic segmentation model for diffuse midline glioma with H3K27M alteration

**DOI:** 10.3389/fonc.2025.1602516

**Published:** 2026-01-12

**Authors:** Yong Deng, Tengyun Chen, Yuekang Zhang, Bowen Huang

**Affiliations:** 1 Department of Neurosurgery, West China Hospital, Sichuan University, Chengdu, Sichuan, China; 2 Department of Cardiovascular Surgery, West China Hospital, Sichuan University, Chengdu, Sichuan, China

**Keywords:** automatic segmentation, deep learning, diffuse midline glioma, glioma, H3K27M alteration

## Abstract

**Background:**

Diffuse midline glioma (DMG) is a fatal tumor that emerges in the brainstem and thalamus. Compared with microsurgery and chemotherapy, radiotherapy is currently regarded as a safer and more effective treatment option. However, mapping radiotherapy target on brain stem is extremely demanding. In the study, we build a deep learning-based diffuse midline glioma with H3K27M alteration radiotherapy target area automatic delineation model.

**Methods:**

We collected contrast-enhanced T1-weighted (T1C), T2-weighted, and T2 fluid attenuated inversion recovery (T2-Flair) sequences from patients with DMG and H3K27M alteration from two medical centers to train and test the model. Based on the framework of generative adversarial networks (GANs), we integrated spatial channel attention mechanism and multi-scale feature extraction according to the characteristics of tumor location in the midline region and diverse morphological changes.

**Results:**

The training and test sets included 116 and 26 patients, respectively. In the training set, the segmentation performance was best for the T2 sequence model, with a Dice similarity coefficient (DSC) of 0.916, followed by the T2-Flair sequence model, with a DSC of 0.893; and the T1ce sequence model had the lowest segmentation accuracy, with a DSC of 0.802. In the test set, the DSC values for the T1C, T2, and T2-Flair sequence models were 0.750, 0.872, and 0.862, respectively, demonstrating the strong generalizability of the model.

**Conclusions:**

We developed DMG with H3K27M alteration automatic segmentation model based on GANs for the first time. It shows excellent automatic segmentation accuracy and generalizability.

## Introduction

Brainstem gliomas (BGs) are a type of primary neuroglial tumor occurring within the brainstem. It can occur in any age group but predominantly affects children (1). Diffuse intrinsic pontine glioma (DIPG) is the most common type of brainstem tumor in children, constituting more than 80% of BGs in this age group (2). DIPG is a highly aggressive and fatal brain tumor. With increasing understanding of DIPG, the World Health Organization (WHO) reclassified this disease as diffuse midline glioma (DMG) in 2016 (3). With advancements in biotechnology and biopsy techniques, research has shown that most DMGs contain the H3K27M mutation (4, 5). This mutation leads to a global loss of H3K27 trimethylation and an increase in oncogenic H3K27 acetylation (6). DMGs with the H3K27M mutation demonstrate poorer prognoses (2, 7). In the 2021 WHO Central Nervous System Tumor Classification (5th edition), the term “H3K27M mutant” was replaced by “H3K27M alteration” (8).

Among all DMG cases, H3K27M alterations occur in approximately 80% of children and 15–60% of adults (9). DMG with an H3K27M alteration is highly malignant and is considered WHO grade IV, regardless of the histological characteristics (10). At present, the range of surgical resection for DMG patients with H3K27M alteration is still controversial (11, 12), and chemotherapy does not achieve satisfactory results (13, 14). Radiotherapy is currently recognized as a treatment that can benefit patient survival. Standard chemotherapy regimens can delay tumor progression for up to 3 months in 70–80% of patients (15–17). However, mapping radiotherapy targets often requires an experienced neurosurgeon or neuroradiologist. There are several disadvantages: 1, this is a time-consuming and labor-intensive task, requiring several minutes to accurately sketch a patient’s magnetic resonance imaging (MRI); 2, Tumor regions delineated by different physicians inevitably exhibit individual differences and heterogeneity, posing challenges to standardized treatment (18).

Computer vision-based automatic segmentation of gliomas represents a current focal point in medical image analysis, supporting enhanced clinical decision-making for gliomas (19). Many studies have achieved good results (20, 21), but they often target the entire class of gliomas. DMG with H3K27M alteration is a highly malignant tumor located in the midline region, and H3K27M alteration can affect the MRI manifestations of the tumor (22). However, there is currently a lack of research on automatic MRI segmentation for DMGs with H3K27M alteration. Therefore, the purpose of this study is to construct an automatic delineation model for the radiotherapy target of a DMG with H3K27M alteration based on deep learning.

## Materials and methods

### Data collection

**Figure 1:**
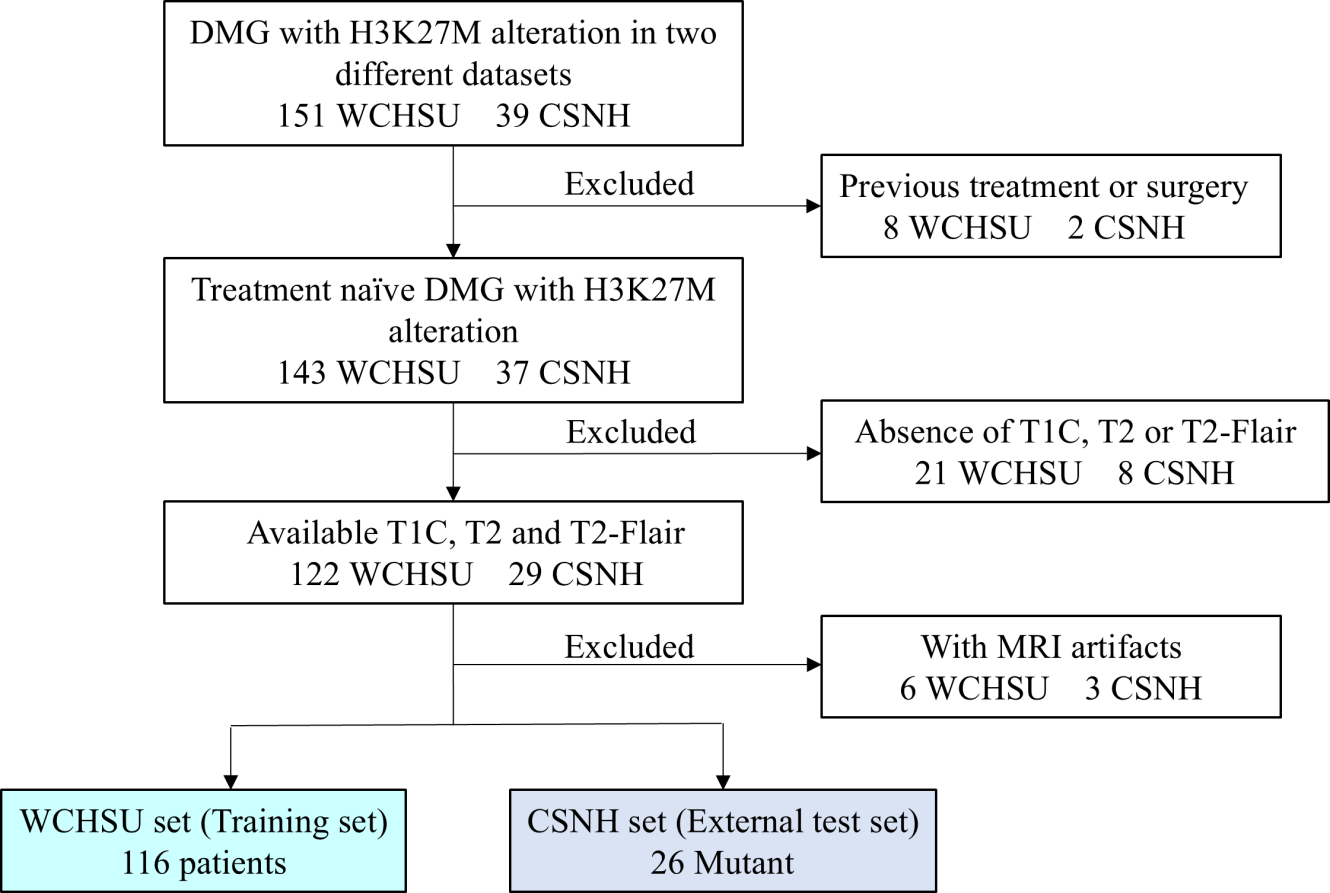
Patient selection flowchart for this study.

We collected data from patients diagnosed with DMG harboring the H3K27M alteration at West China Hospital, Sichuan University (WCHSU), from February 1, 2016, to May 31, 2023, which served as the training set. A similar group of patients from Chengdu Shangjin Nanfu Hospital (CSNH) was collected as the test set during the same period. The exclusion criteria were as follows: (1) patients with a history of surgery, radiotherapy, or chemotherapy prior to the current diagnosis; (2) absence of T1-weighted contrast-enhanced (T1C) or T2-weighted imaging sequences preoperatively; and (3) the presence of artifacts in preoperative MRI scans disrupting image clarity. According to the 2021 National Comprehensive Cancer Network (NCCN) guidelines, abnormalities detected in T1C or T2 fluid-attenuated inversion recovery (T2-Flair) sequences are recommended for defining the gross target volume (GTV) for gliomas. The consensus among Chinese experts on glioma radiotherapy suggests the use of T2 or T2-Flair sequences as the standard for delineating the GTV. Therefore, we collected T1C, T2, and T2-Flair sequences from patients for model training. The patient selection flowchart for this study is presented in **Figure 1**. This study was approved by the Ethics Committee of West China Hospital, Sichuan University (Ethics No. 2023.2064). As this was a retrospective study, patient informed consent was waived by the Ethics Committee of West China Hospital, Sichuan University. Authors had access to information that could identify individual participants during or after data collection.

To address the scarcity of data on DMGs with H3K27M alteration, we utilized training set data from the 2021 Brain Tumor Segmentation (BraTS) dataset (23) for model pretraining, which included MRI scans from 1, 251 patients. The BraTS dataset comprises preoperative MRI scans of glioma patients collected from multiple medical centers. These scans were annotated by one to four experienced physicians, who delineated the tumor regions across four imaging sequences: T1, T1C, T2, and T2-Flair (18).

### MRI preparation

All MRI scans were manually delineated slice-by-slice on axial MR images by a neurosurgeon with five years of experience and subsequently reviewed by neurosurgeons with 10 and 30 years of experience. All annotations were completed via LabelMe software version 3.16.2. To improve segmentation precision, the images were magnified tenfold during the delineation process. MRIs were randomly selected for annotation, and all patient clinical information was concealed. The manually annotated images are displayed in the **Supplementary Figure S1**. After annotation, the images were cropped and resized to 240x240 pixels. To accelerate model convergence, the pixel values were normalized to a range of 0–1. To enhance model generalizability and prevent overfitting, data augmentation techniques such as horizontal flipping and random rotation were applied prior to data input.

### Model construction

**Figure 2:**
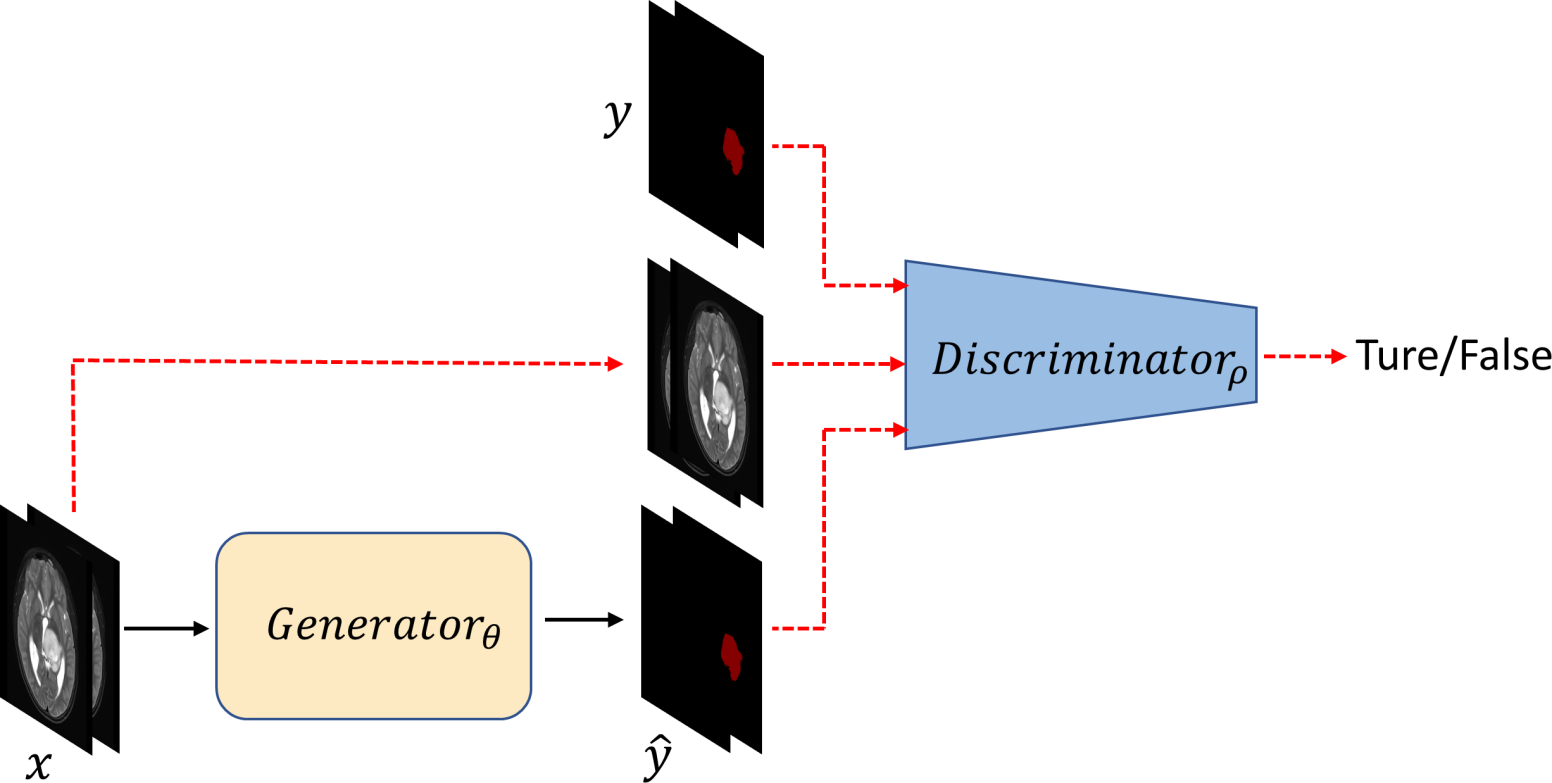
Overall schematic diagram of the model. x represents the unlabeled MRI data; Generator θ refers to the generator module, where θ denotes the parameters of the generator; Discriminator ρ refers to the discriminator module, where ρ denotes the parameters of the discriminator; y represents the ground truth labels; ŷ represents the pseudolabels generated by the generator.

The annotation of medical data is both time-consuming and labor-intensive. Currently, no labeled MRI datasets for DMG with H3K27M alteration are available in public databases, making it challenging to obtain large-scale, finely annotated training data. Insufficient training data often lead to overfitting in CNN models (24). Generative adversarial networks (GANs) are a class of unsupervised learning algorithms derived from zero-sum game theory and consist of a generator and a discriminator. They are designed to estimate the underlying distribution of data samples and generate new data samples (25). Since their inception, GANs have demonstrated superiority in generating realistic images and in solving image-to-image translation problems in natural domains (26, 27). Compared with other models, GANs are notable for completing segmentation tasks with minimal data (28), requiring only 0.8% to 1.6% of the annotated data typically needed by other algorithmic models (29). However, the automatic segmentation of H3K27M-altered tumors using GANs remains unexplored. Therefore, we choose GANs as the foundational framework for this study. A schematic diagram of this study is shown in **Figure 2**. Detailed information about the model’s generator, discriminator, and optimization functions is provided in the **Supplementary Figures S2**, **S3**.

### Module configurations

Given that DMG with H3K27M alteration often occupies the midline position in spatial terms, typically located in the central region of the image, we incorporated the spatial and channel squeeze and excitation (ScSE) module (30) to enable the model to allocate more attention to spatial location information, thereby improving segmentation accuracy. To address the issue where single-scale convolutional layers in neural networks may not always adapt to a wide range of tumor sizes (31), we introduced a pyramid pooling module (32) to facilitate multi-scale feature extraction (MFE). To address the issue of unstable training in GANs, we introduced the convolutional block attention module (CBAM) (33) into the network. This integration aims to increase the stability of GAN training and improve the quality of image generation (34). Detailed schematic diagrams of the ScSE, MFE, and CBAM modules are provided in the **Supplementary Figures S4**-**S6**, respectively.

### Model training

In the experiment, adamW (35) was adopted as the optimizer of the model, and the initial parameters of the model were set as follows: initial learning rate =0.0001, k=0.5, p=2, batch size = 2, and number of epochs = 500. The learning rate is automatically adjusted via cosineAnnealingLR during the training process. The training of the model was divided into two stages. In the first stage, we trained the training set together with the BraST dataset to make the model familiar with the common characteristics of gliomas and capable of automatic segmentation of gliomas. The training adopted 5-fold cross-validation. The second stage involved fine-tuning the model solely with the training set to enhance its segmentation performance for DMGs with H3K27M alteration. Later, its generalizability is tested on the test set. We have detailed the parameter quantities of the generator and discriminator in the **Supplementary Tables S1**, **S2**, respectively.

### Ablation experiment

To verify the effectiveness of the unsupervised learning, MFE, ScSE, and CBAM modules in this study, we designed ablation experiments and trained five models, which are as follows: 1. MSCG, incorporating MFE, ScSE, and CBAM modules into the GAN model; 2. MSCG-Dis, supervised model, with only the first half (generator) of the GAN model, without the discriminator part; 3. SCG, the MFE module has been removed from the MSCG model; 4. MCG, the ScSE module has been removed from the MSCG model; 5. MSG, the CBAM module, has been removed from the MSCG model.

### Evaluation index

We used the Dice similarity coefficient (DSC), 95% Hausdorff distance (HD95) (36), Jaccard similarity coefficient (JSC), sensitivity, and predictive positive value (PPV) to evaluate the performance of the model in automatic segmentation. The DSC indicates the spatial overlap between the segmented MR image automatically generated by the model and the real segmented MR image sketched by the neurosurgeon. HD95 represents the top 95% of all distances between the nearest points in the real label and the pseudolabel. The closer HD95 is to 0, the higher the edge similarity between the two images and the better the model performance. This indicator is more sensitive to the accuracy of edge segmentation. The closer the JSC value is to 1, the better the model performance. When the PPV value is between 0 and 1 and the value is closer to 1, the normal tissue pixels are less likely to be misclassified as tumor tissue pixels.

### Experimental environment and configuration

Our neural network is implemented on an NVIDIA RTX 1080Ti graphics processing unit (GPU), Intel Xeon E5–1650 central processing unit (CPU), and 64G random access memory (RAM) using the PyTorch 1.7 deep learning framework and Python 3.7 programming language.

## Results

Ultimately, we included a total of 116 patients in the training set and 26 patients in the test set. The results of different models on the T1C, T2, and T2-Flair sequences in the training set are presented in **Table 1**. On the T1C sequence, the MSCG model performed the best, with a DSC of 0.802, an HD95 of 11.561 mm, a JSC of 0.751, a sensitivity of 0.837, and a PPV of 0.842. After supervised training, the model’s DSC decreased to 0.757, the HD95 increased to 15.478 mm, and the JSC, sensitivity, and PPV also decreased. When the MFE, ScSE, and CBAM modules were removed individually, both DSC and HD95 showed varying degrees of decline, with the most significant drop in DSC (0.05) occurring when the ScSE module was removed. These results indicate that the MFE, ScSE, and CBAM modules all play important roles in improving model performance, with the ScSE module contributing the most to the enhancement of the model’s performance. The results of different models on the T1C sequence were similar in the T2 and T2-Flair sequences, with the MSCG model demonstrating the best performance across all sequences.

**Table 1:** Automatic segmentation results of the model on T1C, T2, and T2-flair sequences in the training set.

Models	MRI	DSC	HD95 (mm)	JSC	Sensitivity	PPV
MSCG	T1C	**0.802**	**11.561**	**0.751**	**0.837**	**0.842**
	T2	**0.916**	**4.879**	**0.845**	**0.923**	**0.891**
	T2-Flair	**0.893**	**6.347**	**0.813**	**0.902**	**0.916**
MSCG-Dis	T1C	0.757	15.478	0.723	0.795	0.825
	T2	0.835	6.571	0.776	0.857	0.834
	T2-Flair	0.831	8.953	0.736	0.847	0.873
SCG	T1C	0.761	13.827	0.716	0.802	0.813
	T2	0.873	6.334	0.817	0.892	0.873
	T2-Flair	0.858	7.759	0.764	0.865	0.868
MCG	T1C	0.752	14.352	0.687	0.786	0.807
	T2	0.848	7.526	0.793	0.901	0.851
	T2-Flair	0.821	7.501	0.723	0.849	0.861
MSG	T1C	0.773	12.896	0.732	0.819	0.821
	T2	0.882	5.638	0.825	0.915	0.868
	T2-Flair	0.853	7.287	0.782	0.872	0.893

Bold values indicate the best performance among the five models; DSC, Dice Similarity Coefficient; HD95, 95% Hausdorff Distance; JSC, Jaccard Similarity Coefficient; PPV, Predictive Positivity Value.

A comparison of the segmentation accuracy of the model across different MRI sequences revealed that the T2 sequence model yielded the best results, with a DSC of 0.916. The T2-Flair sequence model achieved a DSC of 0.893, whereas the T1ce sequence presented the lowest segmentation accuracy, with a DSC of 0.802. Although the T2 sequence model had a higher DSC than did the T2-Flair sequence model, its PPV was lower than that of the T2-Flair sequence model. The results of the model on the test set are presented in **Table 2**. We observed that the highest DSC was still achieved by the T2 sequence model, with a DSC of 0.872. However, compared with the training set, the model’s performance slightly decreased across the test set. Specifically, the DSCs for the T1ce, T2, and T2-Flair sequence models decreased by 5.2%, 4.4%, and 3.1%, respectively. The T2-Flair sequence model demonstrated the best generalization performance.

**Table 2:** Automatic segmentation results of the MSCG model on the test set.

MRI	DSC	HD95 (mm)	JSC	Sensitivity	PPV
T1C	0.750	13.156	0.716	0.782	0.818
T2	**0.872**	**6.259**	**0.816**	**0.883**	**0.867**
T2-Flair	0.862	7.237	0.765	0.826	0.832

Bold values indicate the best performance among the three sequences; DSC, Dice Similarity Coefficient; HD95, 95% Hausdorff Distance; JSC, Jaccard Similarity Coefficient; PPV, Predictive Positivity Value.

### Visualization of the segmentation results

**Figure 3:**
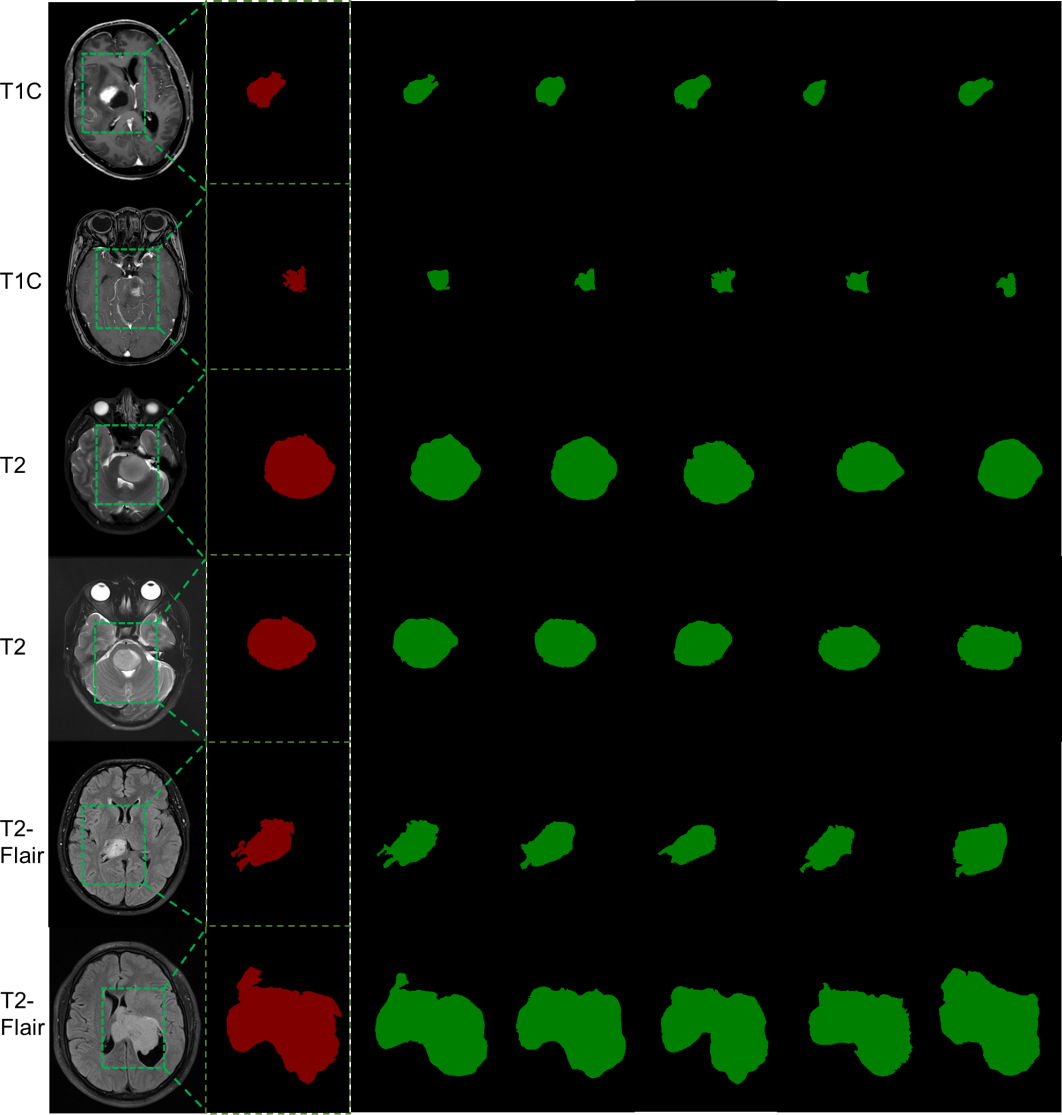
Automatic segmentation results of the model in the training set. The first column displays the original images from different MRI sequences before segmentation, the second column shows the manually segmented images after zooming in on the local ROI, the third column presents the automatic segmentation results from the MSCG model, the fourth column shows the automatic segmentation results from the MSCG-Dis model, the fifth column displays the automatic segmentation results from the SCG model, the sixth column shows the automatic segmentation results from the MCG model, and the seventh column presents the automatic segmentation results from the MSG model.

**Figure 3** presents selected automatic segmentation results generated by the model. The MSCG model’s segmentation outcomes are the most closely aligned with those delineated by experts across the T1C, T2, and T2-Flair sequences. Overall, all the models successfully identified the primary location of the tumor and achieved effective gross segmentation. When finer boundary details are examined, the MSCG model’s segmentation closely resembles the expert-delineated edges, demonstrating greater precision. In contrast, the segmentation boundaries produced by the MSCG-Dis, SCG, MCG, and MSG models deviate further from the expert segmentation boundaries and appear to be relatively coarse. These findings further support the notion that unsupervised learning, along with the MFE, ScSE, and CBAM modules, significantly enhances the model’s segmentation accuracy, with the MSCG model exhibiting the best performance.

**Figure 4:**
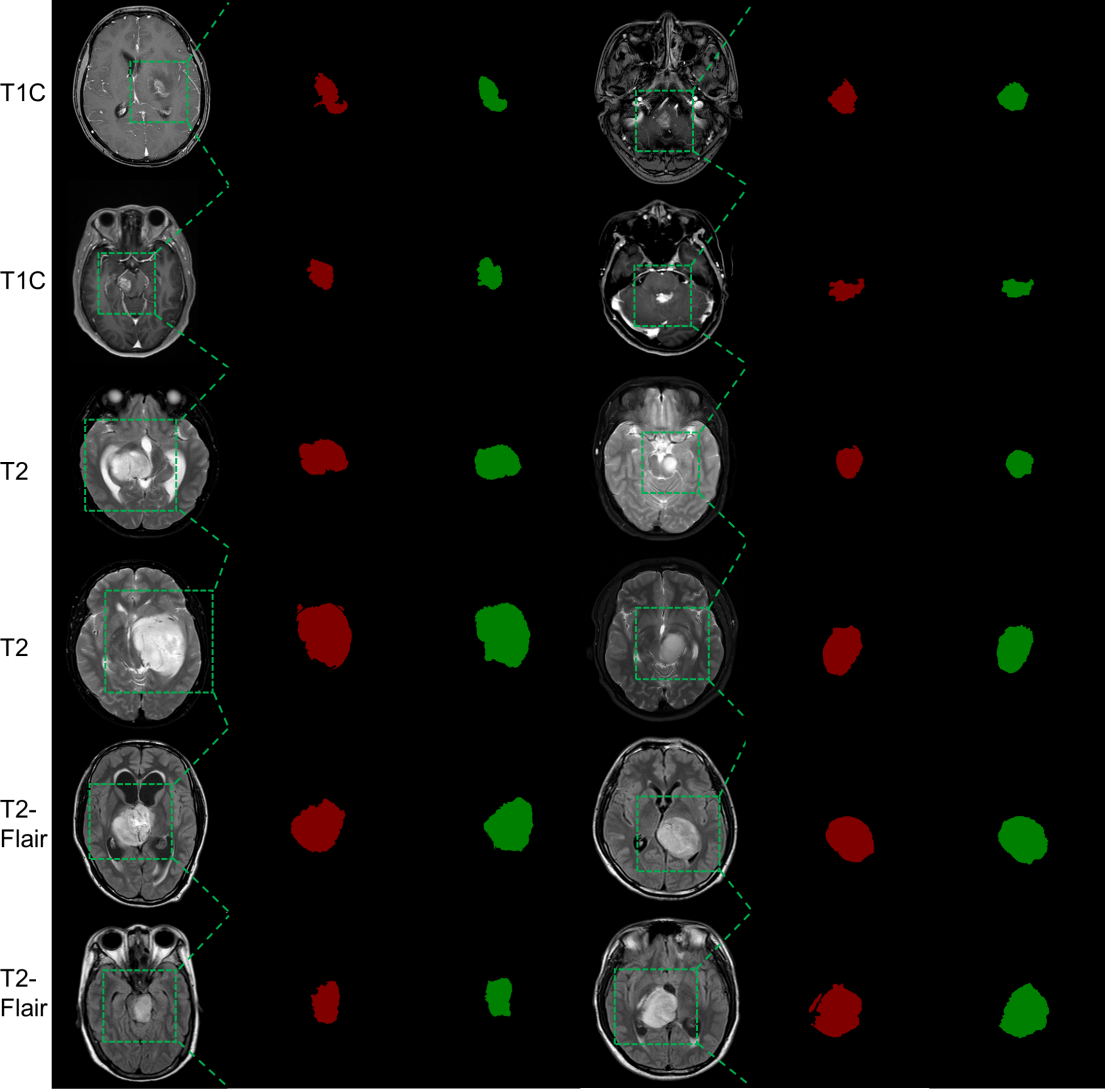
Automatic segmentation results of the MSCG Model on the test set. The red areas indicate manually segmented labels, while the green areas represent the automatic segmentation results.

In **Figure 4**, we present the automatically segmented images generated by the MSCG model on the test set. We observed that all three sequences achieved relatively accurate overall contour localization. In the T1C sequence, the regions of interests (ROIs) exhibit complex morphologies, including branches, and generally cover relatively small areas. In contrast, the ROIs in the T2 and T2-Flair sequences are more rounded, with fewer branches and larger areas. This difference in morphology may be one of the reasons why the model performs better on the T2 and T2-Flair sequences than on the T1C sequence.

## Discussion

In this study, we developed the first automatic segmentation model specifically targeting a DMG with H3K27M-positive molecular markers. To address the common challenge of limited medical data, we included 1, 251 glioma patients from the BraTS dataset in the first phase of model training. Additionally, we employed a GAN network, known for achieving strong performance even with small datasets, as the foundational framework for this study. Considering the characteristic location of the DMG with H3K27M alteration (typically along the midline), we incorporated the ScSE module into the model. To fully capture both local and global features in MR images, we also integrate the MFE module. Ablation experiments were designed to assess the contribution of each module to the model’s performance. The final model achieved excellent results and demonstrated strong generalizability on the test set.

The model exhibited the best segmentation performance on the T2 sequence, with all the metrics surpassing those of the T2-Flair sequence. However, the PPV for the T2 sequence was lower than that for the T2-Flair sequence, indicating a greater likelihood of misclassifying normal tissue as tumor tissue in the T2 sequence. This may be due to the location of the DMG near the midline, close to the ventricles, where cerebrospinal fluid (CSF) signals in the ventricles closely resemble tumor signals in the T2 sequence, leading the model to misinterpret CSF as tumor tissue. In contrast, the T2-Flair sequence shows low signal intensity for CSF and high signal intensity for edema caused by the tumor, making it easier to distinguish between the two and reducing the likelihood of misclassification. The segmentation performance on the T1C sequence was inferior to that of the other two sequences, likely because gliomas almost always present with abnormally high signals in the T2 or T2-Flair sequences but may not exhibit high signals in the T1C sequence, resulting in fewer T1C sequences available for training and consequently lower model performance than the other two sequences do.

Ablation experiments demonstrated that the modules we introduced, along with the use of unsupervised learning, significantly improved the model’s performance. Compared with the other modules, the ScSE module provided the most significant improvement in the model’s automatic segmentation performance for the T1C and T2-Flair sequences. Previous studies on automatic glioma segmentation have also shown that the ScSE module exhibits strong performance (37, 38). In deep learning, attention mechanisms are often employed to focus on important information while disregarding less relevant data (39). The ScSE module likely enhances model performance by assigning different parameter weights to key regions of the image, thereby directing the model’s attention to the lesion areas and improving segmentation accuracy.

In our study, the size and shape of the DMG with H3K27M alteration tumors varied significantly, prompting us to use the MFE module to enhance the model’s ability to extract multiscale and multilevel information. Previous studies on automatic glioma segmentation have also demonstrated the effectiveness of pyramid pooling in multi-scale feature extraction (40). In our research, by comparing the results of the MSCG and MSCG-Dis models in ablation experiments, we found that the GAN network outperformed the supervised learning models. In earlier studies, Carver et al. (41) explored the use of GANs to augment data, address the scarcity of medical data and further improve the performance of automatic glioma segmentation. Their results showed that GANs could synthesize high-quality MR images, highlighting the excellent data generation capabilities of GANs—a conclusion supported by our research as well.

This study has several limitations. First, the dataset is relatively small, consisting of only 116 training and 26 test cases from two centers—a common challenge in rare tumor studies but one that may affect model robustness. We partially addressed this by incorporating the BraTS dataset and employing a GAN framework known for performing well with limited data, and future work will require larger multi-center datasets. Second, the model’s generalizability across institutions needs further validation, as differences in scanners and imaging protocols may impact performance. Third, the study lacks multimodal and longitudinal validation, limiting its ability to capture disease progression or integrate complementary imaging information. Finally, due to the scarcity of existing research on automated segmentation of H3K27M-altered DMG, comparative analysis with other methods remains limited and will be essential as the field advances.

## Conclusion

We developed the first end-to-end automatic segmentation model for DMG with H3K27M alteration based on a GAN network. By pretraining on a large dataset and incorporating spatial-channel attention mechanisms and multi-scale feature extraction algorithms, which are tailored to the tumor’s central location and significant size variation, the model achieves excellent automatic segmentation results. The model also demonstrated strong generalization performance on the test set. We hope that this work will provide a novel reference for delineating radiotherapy target areas in DMG with H3K27M alteration in the future.

## Data Availability

The original contributions presented in the study are included in the article/**Supplementary Material**. Further inquiries can be directed to the corresponding author.

## References

[1] Kuzan-Fischer CM Souweidane MM . The intersect of neurosurgery with diffuse intrinsic pontine glioma. J Neurosurg Pediatr. (2019) 24:611–21. doi: 10.3171/2019.5.PEDS18376, PMID: 31786541

[2] Vanan MI Eisenstat DD . DIPG in Children - What Can We Learn from the Past? Front Oncol. (2015) 5:237. doi: 10.3389/fonc.2015.00237, PMID: 26557503 PMC4617108

[3] Louis DN Perry A Reifenberger G Von Deimling A Figarella-Branger D Cavenee WK . The 2016 World Health Organization Classification of Tumors of the Central Nervous System: a summary. Acta Neuropathol. (2016) 131:803–20. doi: 10.1007/s00401-016-1545-1, PMID: 27157931

[4] Schwartzentruber J Korshunov A Liu XY Jones DT Pfaff E Jacob K . Driver mutations in histone H3.3 and chromatin remodelling genes in paediatric glioblastoma. Nature. (2012) 482:226–31. doi: 10.1038/nature10833, PMID: 22286061

[5] Wu G Broniscer A McEachron TA Lu C Paugh BS Becksfort J . Somatic histone H3 alterations in pediatric diffuse intrinsic pontine gliomas and non-brainstem glioblastomas. Nat Genet. (2012) 44:251–3. doi: 10.1038/ng.1102, PMID: 22286216 PMC3288377

[6] Krug B De Jay N Harutyunyan AS Deshmukh S Marchione DM Guilhamon P . Pervasive H3K27 Acetylation Leads to ERV Expression and a Therapeutic Vulnerability in H3K27M Gliomas. Cancer Cell. (2019) 35:782–97.e8. doi: 10.1016/j.ccell.2019.04.004, PMID: 31085178 PMC6521975

[7] Enomoto T Aoki M Hamasaki M Abe H Nonaka M Inoue T . Midline Glioma in Adults: Clinicopathological, Genetic, and Epigenetic Analysis. Neurologia Med-Chir. (2020) 60:136–46. doi: 10.2176/nmc.oa.2019-0168, PMID: 31902873 PMC7073699

[8] Louis DN Perry A Wesseling P Brat DJ Cree IA Figarella-Branger D . The 2021 WHO Classification of Tumors of the Central Nervous System: a summary. Neuro Oncol. (2021) 23:1231–51. doi: 10.1093/neuonc/noab106, PMID: 34185076 PMC8328013

[9] Schulte JD Buerki RA Lapointe S Molinaro AM Zhang Y Villanueva-Meyer JE . Clinical, radiologic, and genetic characteristics of histone H3 K27M-mutant diffuse midline gliomas in adults. Neuro-Oncol Adv. (2020) 2:vdaa142. doi: 10.1093/noajnl/vdaa142, PMID: 33354667 PMC7739048

[10] Agarwal P Aiyer HM . Diffuse midline glioma-H3K27M mutant. A novel entity with a defining and specific IHC marker. Indian J Pathol Microbiol. (2021) 64:351–3. doi: 10.4103/IJPM.IJPM_287_20, PMID: 33851633

[11] Argersinger DP Rivas SR Shah AH Jackson S Heiss JD . New Developments in the Pathogenesis, Therapeutic Targeting, and Treatment of H3K27M-Mutant Diffuse Midline Glioma. Cancers. (2021) 13:5280. doi: 10.3390/cancers13215280, PMID: 34771443 PMC8582453

[12] Wierzbicki K Ravi K Franson A Bruzek A Cantor E Harris M . Targeting and Therapeutic Monitoring of H3K27M-Mutant Glioma. Curr Oncol Rep. (2020) 22:19. doi: 10.1007/s11912-020-0877-0, PMID: 32030483 PMC7501595

[13] Abe H Natsumeda M Kanemaru Y Watanabe J Tsukamoto Y Okada M . MGMT Expression Contributes to Temozolomide Resistance in H3K27M-Mutant Diffuse Midline Gliomas and MGMT Silencing to Temozolomide Sensitivity in IDH-Mutant Gliomas. Neurologia Med-Chir. (2018) 58:290–5. doi: 10.2176/nmc.ra.2018-0044, PMID: 29848907 PMC6048353

[14] Guerra-García P Marshall LV Cockle JV Ramachandran PV Saran FH Jones C . Challenging the indiscriminate use of temozolomide in pediatric high-grade gliomas: A review of past, current, and emerging therapies. Pediatr Blood Cancer. (2020) 67:e28011. doi: 10.1002/pbc.28011, PMID: 31617673

[15] Cohen KJ Broniscer A Glod J . Pediatric glial tumors. Curr Treat Opt Oncol. (2001) 2:529–36. doi: 10.1007/s11864-001-0074-9, PMID: 12057098

[16] Johung TB Monje M . Diffuse Intrinsic Pontine Glioma: New Pathophysiological Insights and Emerging Therapeutic Targets. Curr Neuropharmacology. (2017) 15:88–97. doi: 10.2174/1570159X14666160509123229, PMID: 27157264 PMC5327455

[17] Long W Yi Y Chen S Cao Q Zhao W Liu Q . Potential New Therapies for Pediatric Diffuse Intrinsic Pontine Glioma. Front Pharmacol. (2017) 8:495. doi: 10.3389/fphar.2017.00495, PMID: 28790919 PMC5525007

[18] Menze BH Jakab A Bauer S Kalpathy-Cramer J Farahani K Kirby J . The Multimodal Brain Tumor Image Segmentation Benchmark (BRATS). IEEE Trans Med Imaging. (2015) 34:1993–2024. doi: 10.1109/TMI.2014.2377694, PMID: 25494501 PMC4833122

[19] Liu XB Hou SF Liu S Ding WP Zhang YD . Attention-based multimodal glioma segmentation with multi-attention layers for small-intensity dissimilarity. J King Saud Univ-Comput Inf Sci. (2023) 35:183–95. doi: 10.1016/j.jksuci.2023.03.011

[20] Yang T Song J Li L Tang Q . Improving brain tumor segmentation on MRI based on the deep U-net and residual units. J X-ray Sci Technol. (2020) 28:95–110. doi: 10.3233/XST-190552, PMID: 31839620

[21] Zhou Z He Z Shi M Du J Chen D . 3D dense connectivity network with atrous convolutional feature pyramid for brain tumor segmentation in magnetic resonance imaging of human heads. Comput Biol Med. (2020) 121:103766. doi: 10.1016/j.compbiomed.2020.103766, PMID: 32568669

[22] Zhao JP Liu XJ Lin HZ Cui CX Yue YJ Gao S . MRI comparative study of diffuse midline glioma, H3 K27-altered and glioma in the midline without H3 K27-altered. BMC Neurol. (2022) 22:498. doi: 10.1186/s12883-022-03026-0, PMID: 36550486 PMC9773507

[23] Baid U Ghodasara S Mohan S Bilello M Calabrese E Colak E . The RSNA-ASNR-MICCAI BraTS 2021 Benchmark on Brain Tumor Segmentation and Radiogenomic Classification. (2021). doi: 10.48550/arXiv.2107.02314

[24] Saeed AQ Sheikh Abdullah SNH Che-Hamzah J Abdul Ghani AT . Accuracy of Using Generative Adversarial Networks for Glaucoma Detection: Systematic Review and Bibliometric Analysis. J Med Internet Res. (2021) 23:e27414. doi: 10.2196/27414, PMID: 34236992 PMC8493455

[25] Xun S Li D Zhu H Chen M Wang J Li J . Generative adversarial networks in medical image segmentation: A review. Comput Biol Med. (2022) 140:105063. doi: 10.1016/j.compbiomed.2021.105063, PMID: 34864584

[26] Isola P Zhu JY Zhou T Efros AA . (2017). Image-to-Image Translation with Conditional Adversarial Networks, in: 2017 IEEE Conference on Computer Vision and Pattern Recognition (CVPR), 21–26 July 2017. Piscataway, NJ, USA: IEEE. doi: 10.1109/CVPR.2017.632

[27] Yu Z Xiang Q Meng J Kou C Ren Q Lu Y . Retinal image synthesis from multiple-landmarks input with generative adversarial networks. Biomed Eng Online. (2019) 18:62. doi: 10.1186/s12938-019-0682-x, PMID: 31113438 PMC6528202

[28] Goodfellow I Pouget-Abadie J Mirza M Xu B Warde-Farley D Ozair S . Generative adversarial networks. Commun ACM. (2020) 63:139–44. doi: 10.1145/3422622

[29] Lahiri A Jain V Mondal A Biswas PK . (2020). Retinal Vessel Segmentation Under Extreme Low Annotation: A Gan Based Semi-Supervised Approach, in: 2020 IEEE International Conference on Image Processing (ICIP), 25–28 Oct. 2020. Piscataway, NJ, USA: IEEE. doi: 10.1109/icip40778.2020.9190882

[30] Roy AG Navab N Wachinger C eds. Concurrent Spatial and Channel ‘Squeeze & Excitation’ in Fully Convolutional Networks. In: Medical Image Computing and Computer Assisted Intervention. Springer International Publishing, Cham.

[31] Li P Li Z Wang Z Li C Wang M . mResU-Net: multi-scale residual U-Net-based brain tumor segmentation from multimodal MRI. Med Biol Eng Comput. (2023) 62:641–51. doi: 10.1007/s11517-023-02965-1, PMID: 37981627

[32] Zhao H Shi J Qi X Wang X Jia J . (2017). Pyramid Scene Parsing Network, in: 2017 IEEE Conference on Computer Vision and Pattern Recognition (CVPR), 2017 21–26 July. Piscataway, NJ, USA: IEEE. doi: 10.1109/CVPR.2017.660

[33] Woo S Park J Lee J-Y Kweon IS eds. CBAM: Convolutional Block Attention Module. Computer Vision. In: ECCV 2018. Springer International Publishing, Cham.

[34] Ma B Wang X Zhang H Li F Dan J eds. CBAM-GAN: Generative Adversarial Networks Based on Convolutional Block Attention Module. In: Artificial Intelligence and Security, vol. 2019 . Springer International Publishing, Cham.

[35] Loshchilov I Hutter F . (2017). Decoupled Weight Decay Regularization, in: Proceedings of the 7th International Conference on Learning Representations (ICLR 2019). New Orleans, Louisiana, USA: ICLR. doi: 10.1109/CVPR.2017.660

[36] Huttenlocher DP Klanderman GA Rucklidge WJ . Comparing images using the Hausdorff distance. IEEE Trans Pattern Anal Mach Intell. (1993) 15:850–63. doi: 10.1109/34.232073

[37] Su R Liu JH Zhang DY Cheng CD Ye MQ . Multimodal Glioma Image Segmentation Using Dual Encoder Structure and Channel Spatial Attention Block. Front Neurosci. (2020) 14. doi: 10.3389/fnins.2020.586197, PMID: 33192272 PMC7655917

[38] Tripathi PC Bag S . An Attention-Guided CNN Framework for Segmentation and Grading of Glioma Using 3D MRI Scans. IEEE-ACM Trans Comput Biol Bioinf. (2023) 20:1890–904. doi: 10.1109/TCBB.2022.3220902, PMID: 36350865

[39] Vaswani A Shazeer N Parmar N Uszkoreit J Jones L Gomez AN . Attention is all you need. Proc 31st Int Conf Neural Inf Process Syst Long Beach Calif USA: Curran Assoc Inc. (2017) 30:6000–10. doi: 10.48550/arXiv.1706.03762

[40] Zhang Z Gao S Huang Z . An Automatic Glioma Segmentation System Using a Multilevel Attention Pyramid Scene Parsing Network. Curr Med Imaging. (2021) 17:751–61. doi: 10.2174/1573405616666201231100623, PMID: 33390119

[41] Carver EN Dai ZZ Liang E Snyder J Wen N . Improvement of Multiparametric MR Image Segmentation by Augmenting the Data With Generative Adversarial Networks for Glioma Patients. Front Comput Neurosci. (2021) 14. doi: 10.3389/fncom.2020.495075, PMID: 33584233 PMC7873446

