# Supplementary material for: A deep learning-based automatic segmentation model for diffuse midline glioma with H3K27M alteration
Source: Front Oncol. 2026 Jan 12;15:1602516. doi: 10.3389/fonc.2025.1602516 (PMC12832457; doi:10.3389/fonc.2025.1602516)
Supplement: Supplementary file 1 [file DataSheet1.docx]

**S1 Fig.** Manually annotated MRI images.

**
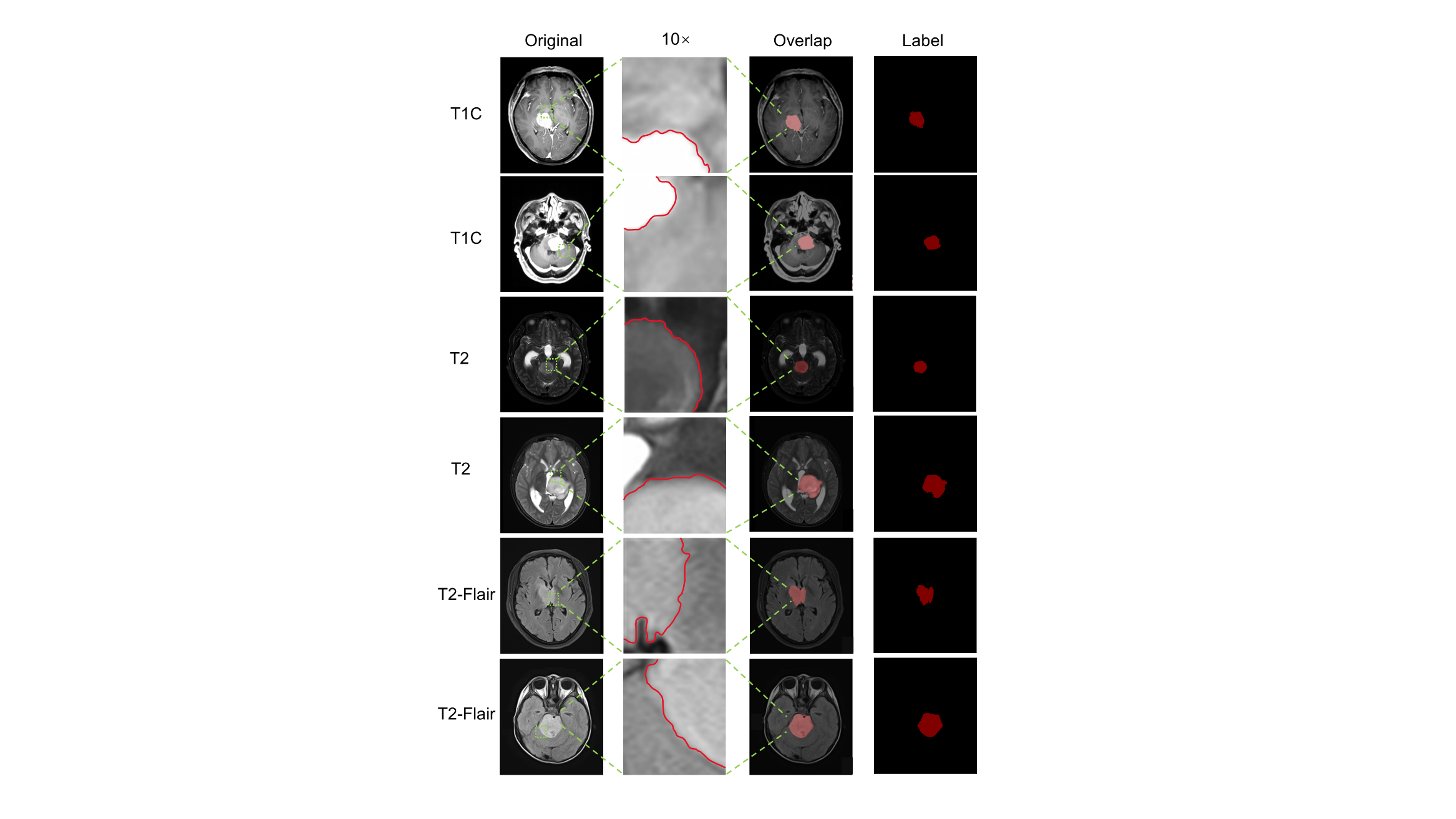
**

The first column shows the original MRI images from the T1C, T2, and T2-Flair sequences. The second column displays the magnified images, zoomed in 10 times, with more precise annotations. The third column presents the overlay of the entire manual segmentation labels on the original images. In the Label column, the regions of interest after manual segmentation are highlighted in red.

**The introduction of the generator for this model**

The generator in this study adopts a U-net architecture, as shown in S2 Fig. The left side of the network is the contracting path, consisting of four layers, each responsible for feature extraction using MFE and ScSE modules, with 2x2 max pooling applied between layers for downsampling. The right side is the expansive path, also comprising four layers, with each layer involving convolution operations followed by ScSE operations, and 2x2 upsampling between layers. There are skip connections between corresponding layers of the contracting and expansive paths to share information. The final output is a two-channel segmentation map.

**S2 Fig.** Structure of the Generator.


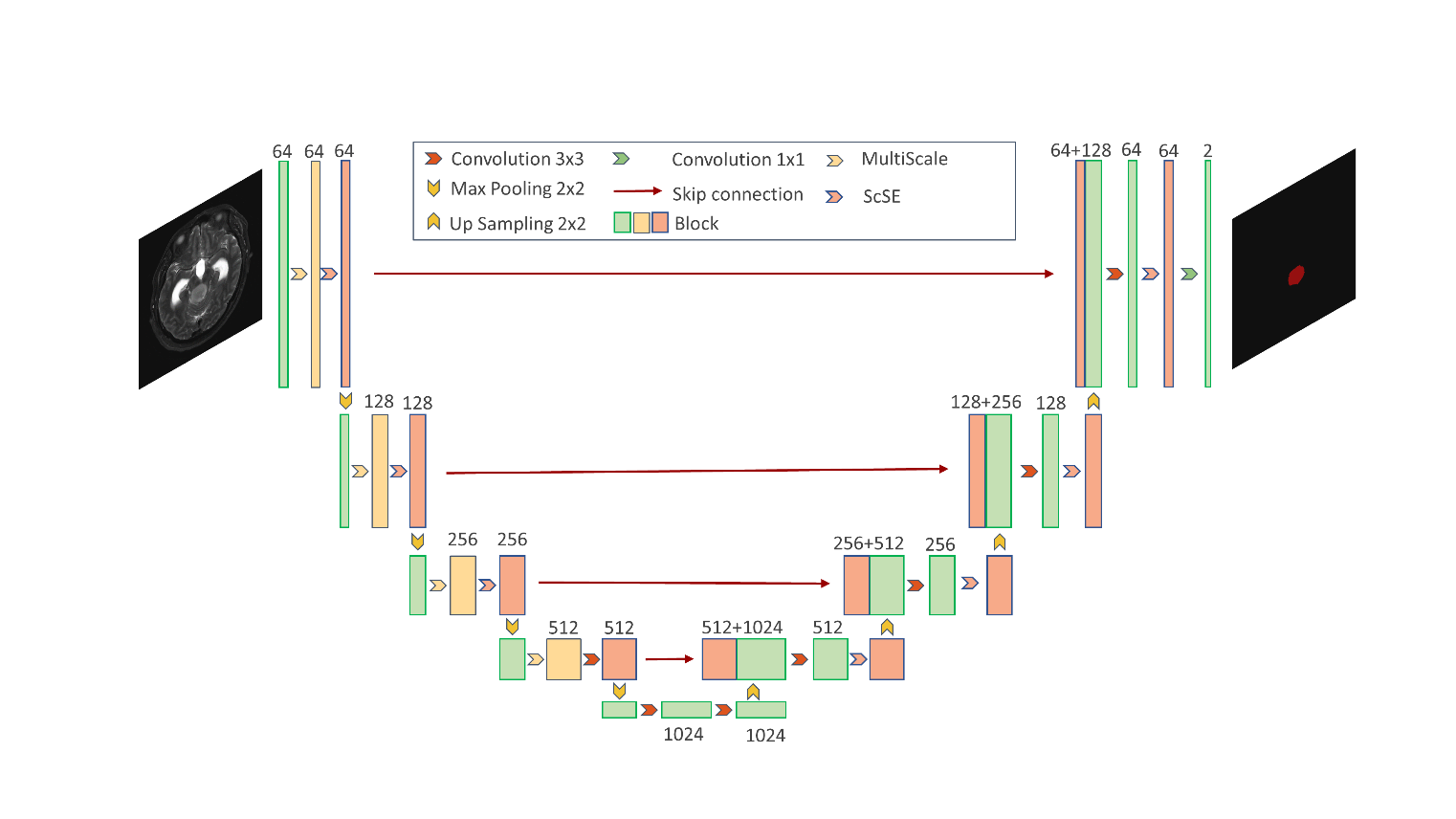


*Convolution 3x3 refers to a 3x3 convolution operation; Convolution 1x1 refers to a 1x1 convolution operation; MultiScale denotes multi-scale feature extraction; Max Pooling 2x2 refers to a 2x2 max pooling operation; Skip connection indicates the skip connection; ScSE represents the spatial and channel squeeze-and-excitation mechanism; Up Sampling 2x2 refers to 2x2 upsampling.

Generator Optimization Function:

$$\underset{\theta}{Min}\left[ \sum_{y^{(j)}\in Y} -D_{\rho}(x^{(j)},G_{\theta}(x^{(j)})，y^{(j)}) \right]$$

Here, *x* represents the unlabeled MRI, *y* represents the manually labeled MRI, *Y* denotes the dataset of manually labeled MRI, and *θ* represents the parameters within the generator.

**The introduction of the discriminator for this model**

In this study, the discriminator, as shown in S3 Fig, consists of four layers. Each layer includes a convolutional pooling module and a CBAM module. The final output is a binary classification result, either T (true label) or F (false label generated by the generator).

**S3 Fig.** Structure of the discriminator.


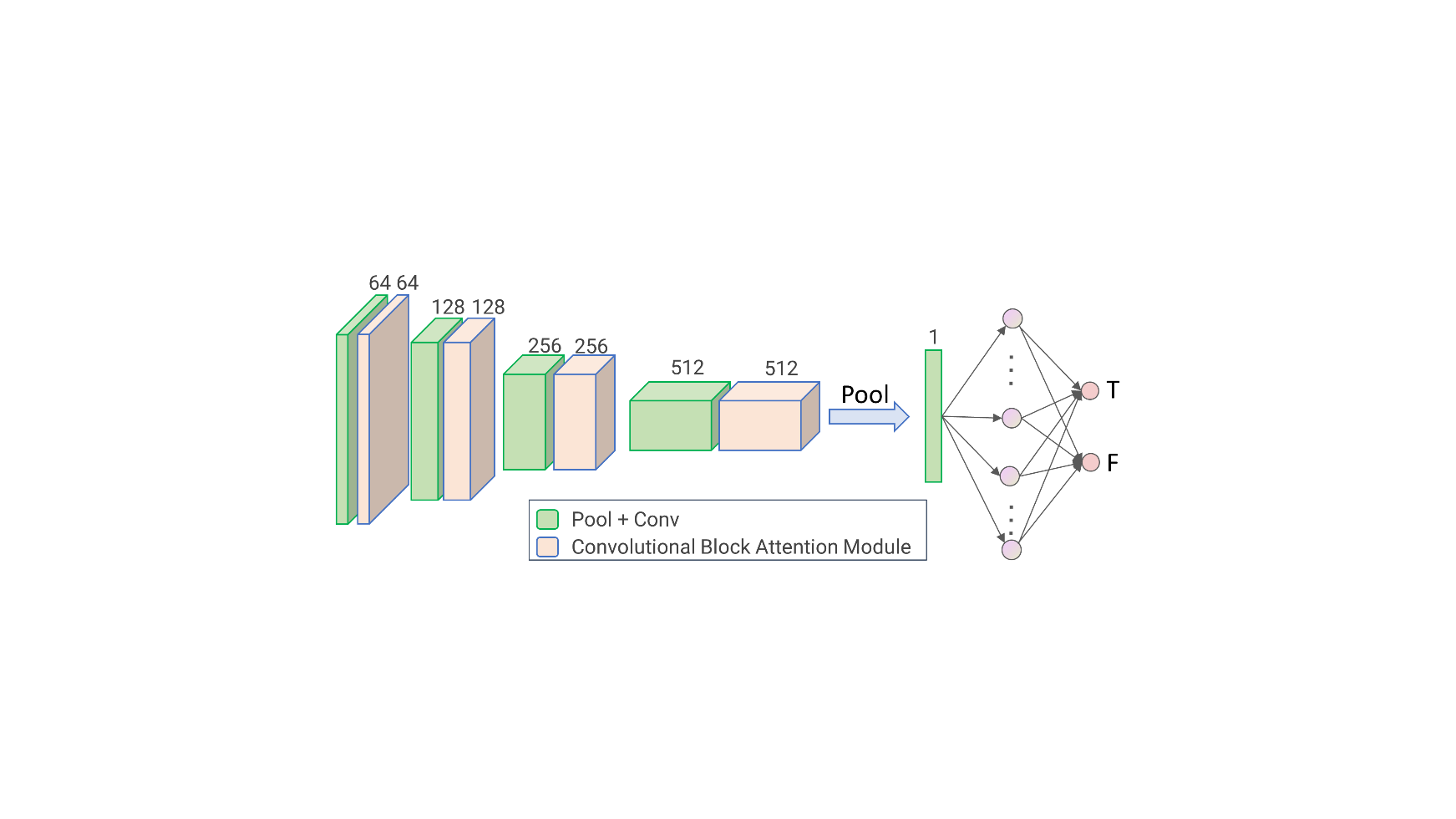


Discriminator Optimization Function:

$${Max}_{\rho\in W}\left[ \sum_{y^{(j)}\in Y,x^{(j)}\in X} D_{\rho}(x^{(j)},y^{(j)},y^{(j)})-\sum_{x^{(j)}\in X,y^{(j)}\in Y} D_{\rho}(x^{(j)},G_{\theta}(x^{(j)}),y^{(j)})+k\left\| \nabla_{\hat{y}^{(j)}}D_{\rho}\left( x^{(j)}，\hat{y}^{(j)}，y^{(j)} \right) \right\|^{p} \right]$$

*x* represents the unlabeled MRI, *X* denotes the unlabeled MRI dataset, *y* represents the manually labeled MRI, and *Y* denotes the manually labeled MRI dataset. *θ* refers to the parameters in the generator, while *ρ* refers to the parameters in the discriminator, both belonging to the parameter space *W*. The purpose of the third part of the equation is to prevent gradient vanishing.

**S4.** The diagram of the ScSE module in this study.


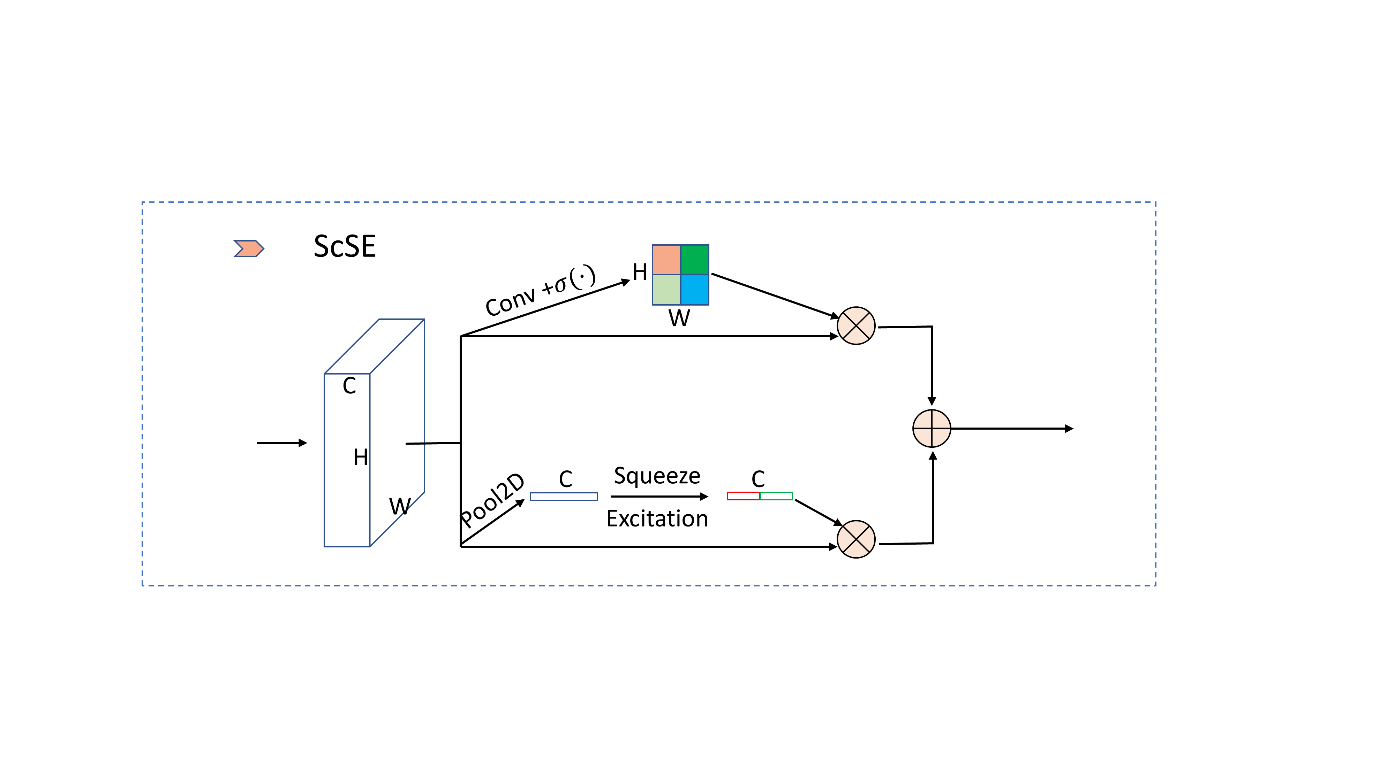


**S5 Fig.** Schematic Diagram of the MFE Module.


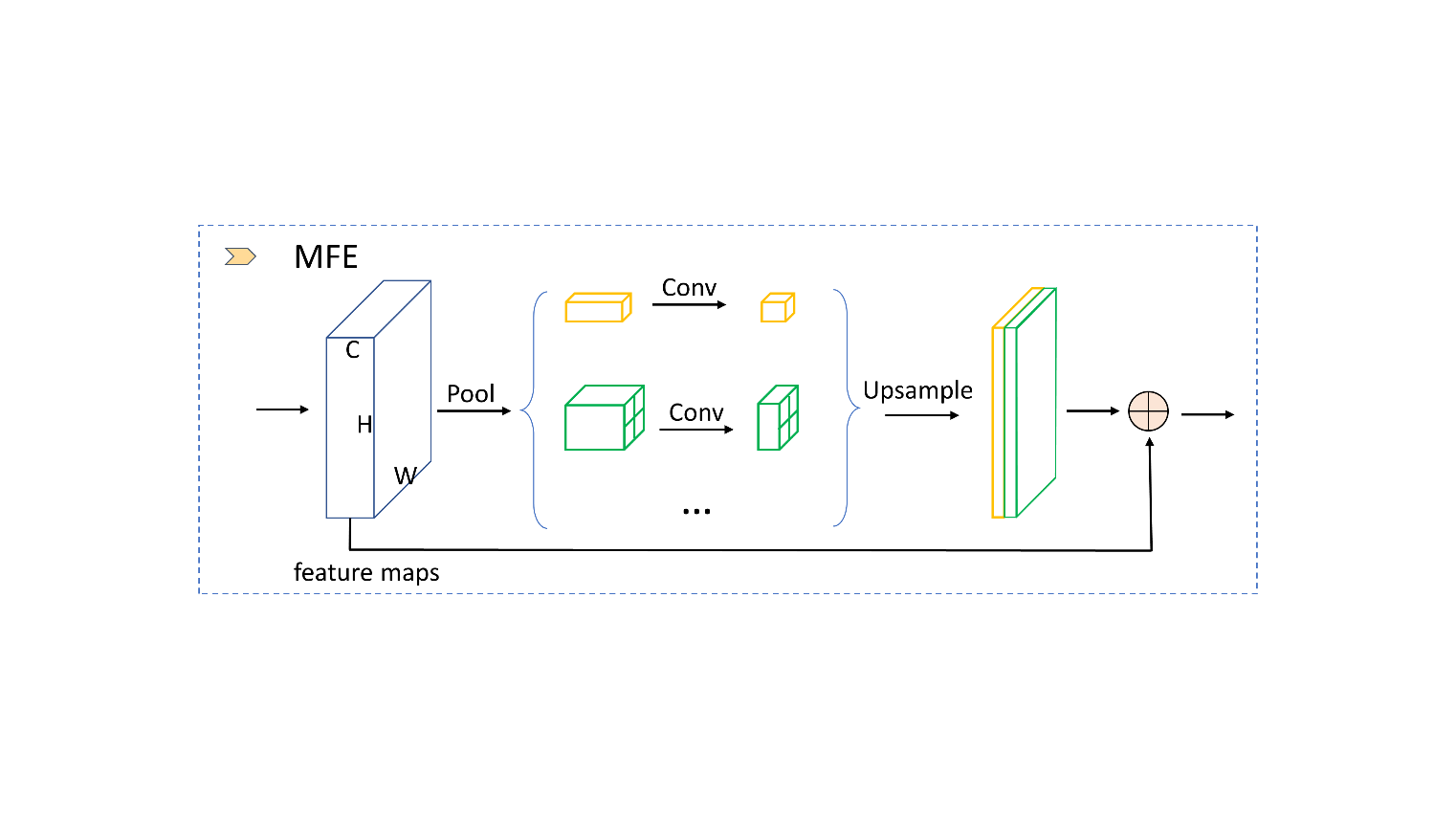


The input is the feature map extracted from the previous convolutional layer, which is then processed through the multi-scale module to capture features at different scales. These are subsequently combined through upsampling and embedding operations to form the final feature map, carrying both local and global contextual information. Finally, this feature map is fed into the next convolutional layer.

**S6 Fig.** Schematic Diagram of the CBAM Module.


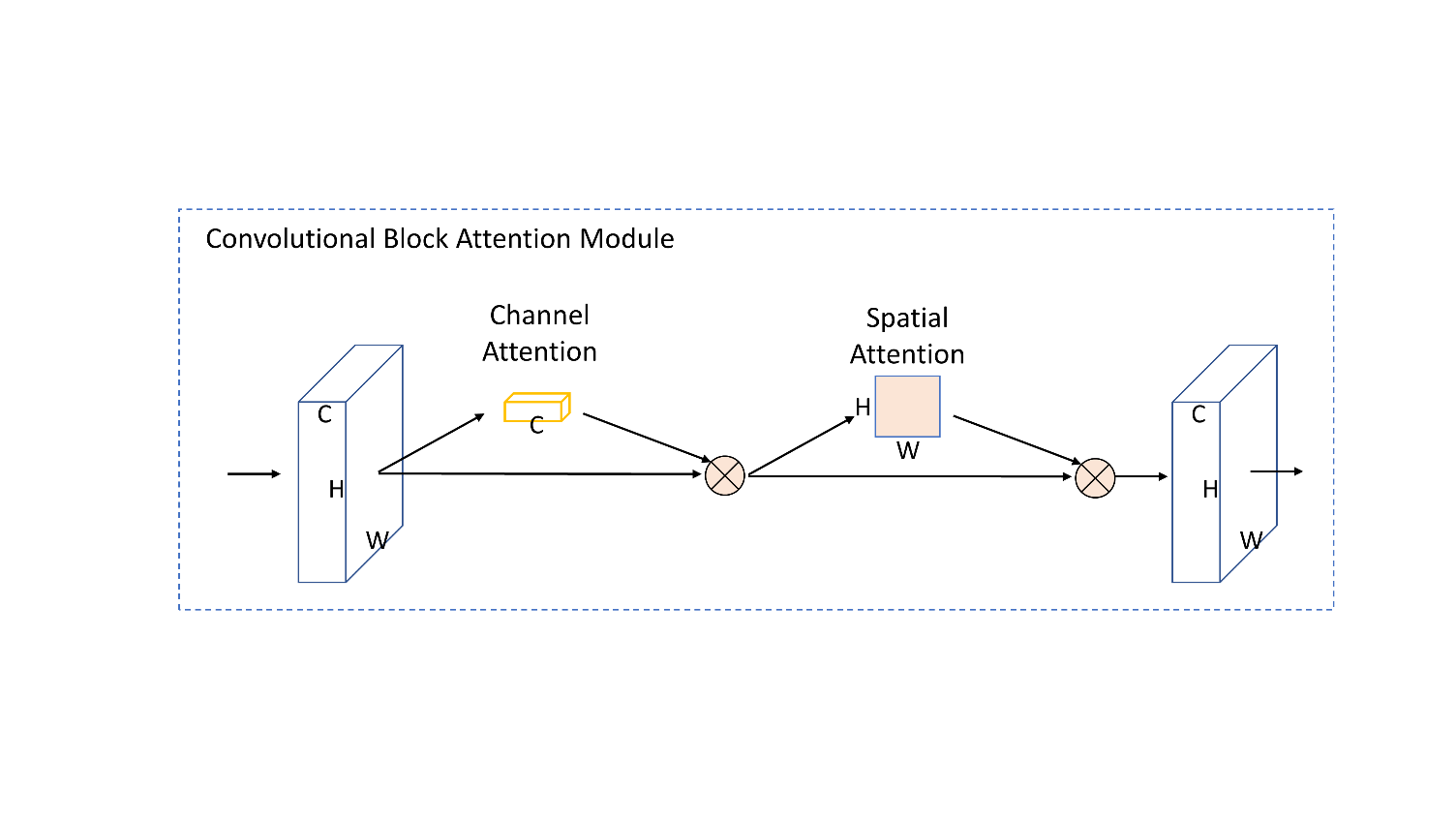


*This module consists of two sub-modules: the channel module and the spatial module. CBAM adaptively refines the intermediate feature maps at each convolutional block in the deep network, enhancing the stability of model training.

**S1 Table.** The number of parameters in the model generator.

| Layer | Operation | Output Channels | Output Feature Size | Training Parameters |
| --- | --- | --- | --- | --- |
| 1.1 | Convolution | 64 | 240*240 | 37824 |
| 1.2 | MFE Module | 64 | 240*240 | 33733 |
| 1.3 | ScSE Module | 64 | 240*240 | 4284 |
| 2.1 | Downsampling | 128 | 120*120 | 221952 |
| 2.2 | MFE Module | 128 | 120*120 | 132997 |
| 2.3 | ScSE Module | 128 | 120*120 | 16705 |
| 3.1 | Downsampling | 256 | 60*60 | 886272 |
| 3.2 | MFE Module | 256 | 60*60 | 528133 |
| 3.3 | ScSE Module | 256 | 60*60 | 66177 |
| 4.1 | Downsampling | 512 | 30*30 | 3542016 |
| 4.2 | MFE Module | 512 | 30*30 | 2104837 |
| 4.3 | ScSE Module | 512 | 30*30 | 263425 |
| 5.1 | Downsampling | 512 | 15*15 | 4721664 |
| 6.1 | Upsampling | 256 | 30*30 | 5900544 |
| 6.2.1 | Channel Attention | 256 | - | 65920 |
| 6.2.2 | Spatial Attention | 1 | 30*30 | 257 |
| 7.1 | Upsampling | 128 | 60*60 | 1475712 |
| 7.2.1 | Channel Attention | 128 | - | 16576 |
| 7.2.2 | Spatial Attention | 1 | 60*60 | 129 |
| 8.1 | Upsampling | 64 | 120*120 | 369216 |
| 8.2.1 | Channel Attention | 64 | - | 4192 |
| 8.2.2 | Spatial Attention | 1 | 120*120 | 65 |
| 9.1 | Upsampling | 64 | 240*240 | 110976 |
| 9.2.1 | Channel Attention | 64 | - | 4192 |
| 9.2.2 | Spatial Attention | 1 | 240*240 | 65 |
| 10 | Convolution | 1 | 240*240 | 65 |

The generator has a total of 20,507,901 training parameters; MFE, Multi-scale Feature Extraction; ScSE, Spatial and Channel Squeeze and Excitation

**S2 Table.** The number of parameters in the model discriminator.

| Layer | Operation | Output Channels | Output Feature Size | Training Parameters |
| --- | --- | --- | --- | --- |
| 1.1 | Convolution | 64 | 240*240 | 38976 |
| 1.2 | CBAM | 64 | 240*240 | 680 |
| 2.1 | Downsampling | 128 | 120*120 | 221952 |
| 2.2 | CBAM | 128 | 120*120 | 2284 |
| 3.1 | Downsampling | 256 | 60*60 | 886272 |
| 3.2 | CBAM | 256 | 60*60 | 8564 |
| 4.1 | Downsampling | 512 | 30*30 | 3542016 |
| 4.2 | CBAM | 512 | 30*30 | 33412 |
| 5.1 | Downsampling | 512 | 15*15 | 4721664 |
| 6.1 | Pooling | 512 | 1*1 | - |
| 7.1 | Linear Layer | 2 | - | 1026 |

The discriminator has a total of 9,456,846 training parameters; CBAM, convolutional block attention module.
